# Drivers and Consequences of ChatGPT Use in Higher Education: Key Stakeholder Perspectives

**DOI:** 10.3390/ejihpe13110181

**Published:** 2023-11-09

**Authors:** Ahmed M. Hasanein, Abu Elnasr E. Sobaih

**Affiliations:** 1 College of Business Administration, King Faisal University, Al-Ahsa P.O. Box 400, Saudi Arabia; 2 Faculty of Tourism and Hotel Management, Helwan University, Cairo P.O. Box 12612, Egypt

**Keywords:** ChatGPT, AI, higher education, chatbot, holistic perspective, drivers, consequences

## Abstract

The incorporation of artificial intelligence (AI) into education has heralded a transformative era in the way students learn and faculties teach. Among the burgeoning array of AI tools, ChatGPT stands out as a versatile and powerful resource. Developed by OpenAI, ChatGPT is an AI-driven conversational model that generates human-like responses. This research draws on the Constructivism Learning Theory to uncover the key drivers pushing higher education students to use ChatGPT for academic purposes, and the multifaceted consequences it brings to the academic environment, by integrating the perspectives of key stakeholders: students, faculty, and education experts/leaders. The key findings of in-depth, face-to-face, interviews with key stakeholders revealed 12 main drivers that motivate students and their faculty to use ChatGPT mainly for learning purposes. However, the findings identified the multifaceted (six positive and another six negative) consequences of using ChatGPT for academic purposes. Recommendations for mitigating the negative consequences of ChatGPT were discussed with key stakeholders, particularly education experts/leaders, who were more concerned about using ChatGPT for academic reasons. The research reveals that higher education institutions should establish clear guidelines as a part of higher education policy, supplemented with training sessions for students and their faculty, about the responsible use of ChatGPT for academic purposes to mitigate any ethical concerns.

## 1. Introduction

Due to the fast-changing environment of higher education, technological advances are continually transforming education for both students and faculty members [1,2]. The emergence of generative artificial intelligence (GenAI) has been recognized as an innovative force, with chat-generative pre-trained transformer (ChatGPT) at the cutting edge of this technological revolution [2,3]. ChatGPT, which is driven by the combination of language processing and machine learning, is an innovative technology that has made its way into education institutions [3]. It is a massive language generator that develops human-like texts in response to a specific command [4]. It could handle a wide range of tasks related to natural language processing (e.g., conversations, translations, and completing texts) [5]. On one hand, several research studies [6,7,8,9] have highlighted the numerous advantages of using ChatGPT in learning, including improving accessibility by providing students and faculty with 24/7 information accessibility and assistance, promoting the learning experience by offering personalized support, and improving language skills by offering grammar suggestions and vocabulary enhancements. Additionally, it assists students in their preparation of assignments and exams, making research activities, and writing academic articles without being easily identified by plagiarism detection programs, which may raise ethical concerns [6].

On the other hand, there were other prominent key challenges concerning using ChatGPT in education. These challenges include reliability issues, the reliance on biased data [6], the limitation of knowledge to information available up to 2021 [7], relying on inaccurate or fabricated content, including fictitious citations [8], and over-reliance on ChatGPT might have negative impact students’ critical thinking and problem-solving abilities [9]. GhatGPT could represent a threat to academic ethics; the response of higher education institutions (HEIs) varies from those who have raced to enforce full constraints on the usage of ChatGPT [7,8] to those who have begun to support it by posting guidelines on how to deal with AI in an ethical and proper way [9]. Notwithstanding this, the majority of HEIs did not publish any information for their students concerning the use of ChatGPT for academic purposes, regarding whether it is permitted or not for academic purposes [10,11]. 

Growing research (see for instance [5,7,8]) has explored the perceptions of students and faculty concerning the use of ChatGPT for academic purposes in higher education. There is a limited, but growing, body of research [12,13] concerning the key benefits, opportunities and challenges of ChatGPT use in higher education. A review of earlier research has confirmed a lack of research studies that adopted a holistic outlook from the perspectives of key stakeholders regarding the use of ChatGPT for academic purposes. Earlier research examined either the perceptions of students or the perceptions of faculty. Nonetheless, there is a lack of studies that integrate and consolidate the perceptions of key stakeholders, which will be undertaken in the current study. 

This research draws on the Constructivism Learning Theory by highlighting the perceptions and roles of active stakeholders (i.e., students, faculty, and education leaders). It explores emerging information and knowledge that ChatGPT could provide for academic purposes [4,10]. ChatGPT could enable users to build on knowledge and information to generate newer information and enhance their experience. Furthermore, ChatGPT’s tailored responses may enhance this process by capitalizing on previous learning and expertise, and offering tailored ideas for future education [14,15]. This response may assist stakeholders in acknowledging deficiencies, and push them into effective progress [16]. This research makes a new attempt to investigate the holistic perspectives of key stakeholders in higher education regarding the drivers and multifaceted consequences of ChatGPT use for academic reasons in higher education. The research explores the mitigation strategies for handling the negative consequences concerning ChatGPT use for academic purposes in higher education. Hence, the following research questions are suggested:

1. What are the drivers that motivate students and their faculty to use ChatGPT for academic purposes in higher education?
2. What are the positive and negative consequences raised by key stakeholders related to ChatGPT use for academic purposes in higher education?
3. What are the mitigation strategies for overcoming negative consequences and better using ChatGPT as an effective and supportive learning tool?

## 2. Literature Review

### 2.1. The Use of ChatGPT in Higher Education

ChatGPT is considered an innovative, transformative and multifaceted AI that serves as an adaptable resource for both students and faculty in a dynamic learning environment [17]. Students benefit from ChatGPT’s ability to provide instant clarification on complex concepts, assistance with assignments, and access to a wealth of information, enhancing their academic journey [18]. It promotes self-directed learning, allowing students to explore subjects at their own pace and style. In terms of faculty, ChatGPT augments teaching by automating administrative tasks, offering personalized feedback, and providing insights into student needs through data analysis. It enables the faculty to focus on teaching strategies, and fosters innovation when designing curriculums [19]. ChatGPT emerges as a valuable tool that can enrich the learning experience, enhance efficiency, and prepare students for the challenges of a rapidly evolving knowledge-based educational environment [13,17]. However, ChatGPT raises concerns about academic integrity and the development of critical thinking skills. It is essential for educational institutions to strike a balance by providing guidance on responsible use, and emphasizing the importance of independent thought [20]. In addition, the integration of ChatGPT into higher education has the potential to reduce the reliance on faculty and, in turn, diminish opportunities for interpersonal connections and human interaction [7]. To aid students and enhance their writing skills, ChatGPT can perform tasks like identifying grammatical and structural issues in their work while also offering valuable feedback [9]. Moreover, students can receive personalized guidance tailored to their unique writing style, allowing them to pinpoint and focus on specific areas requiring improvement [8]. ChatGPT enables computers to simulate human conversations effectively, enabling accurate responses to user queries and personalization by understanding both user intent and context [6]. This opens up diverse possibilities for students, from delving into computer programming to crafting essays and tackling mathematical problems, all of which are achievable with the assistance of ChatGPT [8,9].

According to the constructivist perspective, education is an active process involving the construction of knowledge influenced by factors such as students’ needs, the learning materials at their disposal, the tools they use, and the overall learning environment [21]. Educators assume a pivotal part in this learning process by addressing students’ requirements, providing appropriate learning resources, and offering supportive tools [22]. In contrast to traditional teaching, which often entails the passive reception of knowledge from educators, the constructivist learning theory underscores the importance of autonomous and active learning [23]. A technology-enhanced constructivist learning environment has demonstrated great effectiveness, particularly concerning students’ access to information and their ability to analyze, interpret, and organize this information to expand their knowledge base. Subsequently, Rasul et al. [3] highlighted the applicability of constructivist theory in the context of information transmission. Aligned with the constructivist approach, the use of technology in the learning process empowers students to take charge of their skills and knowledge, allowing them to deal with gaps in their understanding [21,23]. Consequently, it is evident that ChatGPT, as an AI-powered instrument, offers the possibility to foster a constructivist learning experience. This enables them to experiment with ideas, pose inquiries, and obtain instant feedback, thereby facilitating the construction of their own comprehension and knowledge [3].

### 2.2. Drivers and Consequences of ChatGPT in Higher Education

ChatGPT has attracted substantial interest from educational institutions’ stakeholders worldwide as a revolutionary means of educating and supporting students [16,17,18]. Several educational institutions are investigating ways of integrating this artificial intelligence (AI) approach as part of their educational system, identifying its ability to revolutionize traditional pedagogical techniques, improve student interaction, as well as create specific learning environments [20]. On one hand, ChatGPT can be effectively utilized to improve education when its drawbacks are comprehended. In addition to providing responses to theory-based questions, ChatGPT can also generate ideas for research articles [4,15], albeit students should ensure that the generated responses are reliable before using them. ChatGPT, as an effective communicational tool (i.e., Chatbox), can also offer constructive comments on research articles and foster students’ critical thinking and argumentation [16]. In terms of students’ language skills, ChatGPT can also enhance equality in the educational process by leveling expectations for students from non-native English-speaking backgrounds [1]. Likewise, ChatGPT can be used to assist faculties in generating syllabus material for specific modules, developing materials and educational activities (i.e., supporting self-learning), implementing assessments and evaluations, and supporting the research writing process [24]. It might additionally be employed to improve thoughtful teaching techniques by testing existing evaluation techniques and verifying their content, structure, and abilities, along with GenAI, requiring the faculty to generate artificial intelligent proof assessments resulting in a genuine evaluation of students’ educational outcomes [17,25].

However, a recent study [26] found that accuracy and reliability are considered the most common obstacles faced when using ChatGPT in education. Challenges affiliated with reliability involve depending on biased information (i.e., restricted range of data used) [27,28], the lack of updated information (i.e., updated information stopped in 2021) [29] and providing inaccurate/fake data (i.e., presenting fabricated references) [30]. Moreover, the over-reliance on ChatGPT may have a negative effect on students’ ability to think critically and solve problems [31,32]. Concerning plagiarism, data show that articles written using ChatGPT may avoid detection by traditional plagiarism-checking systems [11,33]. Moreover, a recent study [34] found that laws and regulations regarding copyright and intellectual property rights still apply when using AI-generated content (e.g., ChatGPT). The misuse of information generated by ChatGPT may result negatively in copyright violations [27,35]. 

## 3. Methods

### 3.1. Methods Adopted

This research undertook a qualitative methodology in answering the research questions and fulfilling the research objectives. The qualitative methodology was found to be more suitable than quantitative approach for giving a better insight and gaining in-depth information about the drivers and consequences of ChatGPT use for academic purposes in higher education [36]. This research used the consolidated criteria for reporting qualitative studies (QCREQ) [37]. Qualitative research often focuses on the meanings, experiences and perceptions reported in interviews [36,37]; hence, it does not aim to collect quantitative data or report statistical information. All the guidelines suggested in the QCREQ were followed while undertaking the research, processing the data and reporting the findings. With regard to the data collection methods, the research adopted in-depth, face-to-face interviews [38]. We sought to find answers to the research questions about the perceptions of educational stakeholders (i.e., students, faculty, and education leaders) regarding ChatGPT use in higher education, especially its drivers and various consequences. Four core themes were discussed with the three key stakeholders:

1. Perceptions of ChatGPT use for academic purposes in higher educations;
2. Drivers of ChatGPT use by students and academics for academic purposes in higher education;
3. Consequences, whether positive or negative, of ChatGPT use by students and academics for academic purposes in higher education;
4. Strategies for mitigating the negative consequences of ChatGPT use by students and academics for academic purposes in higher education.

Interviews were conducted with students at public universities in Saudi Arabia. Participants were asked to participate in this study voluntarily, and were informed of the study’s purposes. Data collection in this study began at the beginning of the academic year 2023–2024, i.e., in August 2023, and lasted for one month. The participating interviewees had varied educational experiences, facilitating the gathering of meaningful responses and allowing us to investigate the holistic perspectives concerning the drivers and consequences of using ChatGPT in higher education. All interviews were performed one-to-one, and each interview lasted between half an hour and hour, with an average length of forty-five minutes. Both researchers were present during the interviews. The research supervisor took the responsibility of leading the interviews because he has sufficient experience in undertaking qualitative studies. All the interviews were conducted at the most convenient time reported by the interviewees, with the presence of two researchers.

It is also important to note that the current study followed the ethical guidelines throughout. It was approved by the committee in charge at the institution before undertaking the study. Furthermore, the participants’ identities were protected and there was no personal information collected. The purpose of the research was discussed with them before data collection and they were given the opportunity to withdraw from the study at any stage; however, none of the participants withdrew at any stage of the study. Additionally, codes were used when quoting the information given by the participants.

### 3.2. Participants

Participants were selected purposively through the network of researchers working at public universities in KSA. With regard to the type and number of participants, since the research team were on the Business Administration Major course, they were able to connect with students, academics and leaders in the Management, Accounting, Finance, Economics and Quantitative Methods departments. Interviews were done first with 85 students until data saturation was achieved [39]. There were more male participants (82%) than female (18%), due to the limited accessibility of female students to KSA. These were followed by 32 interviews with faculty members, followed by interviews with 21 education leaders and experts. There was almost equal participation of male and female academics, but we saw the limited participation of female leaders. These leaders were the deans of colleges, vice deans, heads of department and experts in quality assurance. The number of selected respondents was enough to attain saturation of data [39]. All of the participants were recorded after they gave approval. The participants were all notified at the start of the interviews that their information was to be used for the study only. The recorded interviews were transcribed after the interviews in Arabic and/or English depending on the language of the participants. However, the languages of the scripts were reviewed by two bilingual (Arabic/English) professionals to ensure the accuracy of the collected data.

### 3.3. Analysis of the Collected Data

A content and thematic analysis technique has been adopted to analyze the data collected from the interviews. Thematic analysis is considered one of the most effective and widespread approaches to analyzing qualitative research data [36]. The collected data were analyzed under four themes:

1. Stakeholders’ perceptions of ChatGPT use for academic purposes in higher education;
2. Key drivers of ChatGPT use by students and academics for academic purposes in higher education;
3. Consequences of ChatGPT use by students and academics for academic purposes in higher education;
4. Recommendations for overcoming negative consequences of ChatGPT use in higher education.

## 4. Results

The data obtained from the in-depth interviews were evaluated manually using thematic analysis. The findings from all interviews have been integrated together, rather than presenting results from each group of participants, to avoid repeating specific points as well as to provide more clarity regarding the collected data. The following sub-sections present the key findings of the study.

### 4.1. Stakeholders’ Perceptions of Using ChatGPT for Academic Proposes in Higher Education

The participants were asked about their perceptions of ChatGPT use for academic purposes in higher education. The key stakeholders’ perceptions about ChatGPT use for academic purposes vary widely depending on various factors, including the context in which it is used, the specific tasks it is employed for, and individual preferences and experiences. In terms of students’ perceptions, the majority of students have a positive view of ChatGPT use for academic purposes in their higher education. Among their comments:

*“ChatGPT is a magical tool. It helps us in our daily educational activities, e.g., assignments, research work, and homework”.*

(ST-3)

*“I believe ChatGPT is a powerful tool that assist on various tasks through a series of queries and responses”.*

(ST-7)

*“I would consider ChatGPT as an excellent search engine. We can use it for many purposes... The answer generates similar human-like responses”.*

(ST-11)

Faculty responses towards using ChatGPT for academic purposes in higher education were multifaceted. Faculty members reported both positive and negative perceptions about using ChatGPT for teaching and learning. The positive perceptions were related to the beneficial aspects of ChatGPT in searching for information, and assisting in academic content creation and designing the syllabus. The negative perceptions regarded the overreliance on ChatGPT in every single detail in education without human thinking, as demonstrated in the following quotes:

*“In one hand, it is a useful tool in collecting information. It is also useful in data presentation. On the other hand, the over-reliance on ChatGPT is one of the critical negative aspects, because it reduces critical thinking”.*

(FC-2)

*“It has a pros and cons. It is an assistive tool for academics that facilitates them in the educational process. Otherwise, it is not an official or authorized tool by our institutions, and students often misuse it”.*

(FC-5)

With regard to education experts and leaders, the majority of them were more likely to have negative perceptions rather than positive. They mentioned that the use of AI in education should add value to the education. The use of AI, including ChatGPT, should add to the learning outcomes, including knowledge and skills. Hence, the use of ChatGPT for academic purposes must be regulated. The educational institutions have to set guidelines for students about its use in a proper way. Examples of these responses could be seen in the following quotes:

*“This tool does not contribute to several skills such as critical-thinking and problem-solving. However, these skills are critical for students’ career”.*

(EDLE-1)

*“This tool will change students’ attitude negatively through turning on cheating information, lessons and answers without any control from their faculty or institutions”.*

(EDLE-4)

To sum up, the perceptions of all respondents regarding the use of ChatGPT varied according to the perspective of each group of interviewees. Students only see the positive aspects of using ChatGPT in their education, while the faculty mentioned that the ChatGPT tool has both positive and negative aspects. With regard to education leaders, they highlighted mainly the negative aspects of using ChatGPT in higher education.

#### 4.1.1. Key Drivers of Using ChatGPT for Academic Purposes in Higher Education

Key stakeholders were asked about the main motives that push them to use ChatGPT as an educational tool or for academic purposes. A summary of the drivers highlighted by the interviewees is shown in Table 1. All interviewees agreed that the quick response and ease of use ChatGPT are considered the most substantial drivers of utilizing it in education. This is a hallmark of its efficiency in education, manifested through its ability to provide immediate answers and support. It also optimizes tasks and raises efficiency. These quick responses have been proven especially valuable in educational environments, where students receive immediate explanations and resources, and faculty as well as education leaders may conserve their time by streamlining their daily tasks. Furthermore, ChatGPT has the ability to offer a smooth, simple, friendly and understandable interface for students and faculty to input queries, receive responses, and navigate its functionalities. These features not only boost stakeholders’ performance, but also fosters a smooth and simple user experience, making ChatGPT a vital tool in today’s digital age.

All interviewees agreed that the quick response and ease of use ChatGPT are the most substantial drivers of utilizing it in education. This is a hallmark of its efficiency in education, facilitating its ability to provide immediate answers and support. It also optimizes tasks and raises efficiency. These quick responses have been proven especially valuable in educational environments, whereby students receive immediate explanations and resources, and faculty as well as education leaders may conserve their time by streamlining their daily tasks. Furthermore, ChatGPT has the ability to offer a smooth, simple, friendly and understandable interface for students and faculty to input queries, receive responses, and navigate its functionalities. These features not only boost stakeholders’ performance, but also foster a smooth and simple user experience, making ChatGPT a vital tool in today’s digital age.

Another driver of using ChatGPT in education has been raised by students and faculty in relation to classroom and homework assistance. Students commented that ChatGPT is a useful tool that can assist them in doing their homework, assignments, and projects. They can ask questions, seek explanations, and get guidance on how to approach their academic tasks. Moreover, faculty noted that ChatGPT could facilitate lecture preparation, the presentation of case studies about specific topics within the lecture, as well as helping them in teaching through giving clear and simple explanations and examples about specific topics, which make them understandable for students.

Language proofreading and editing is considered one of the key drivers that has been cited by both students and faculty for using ChatGPT in education. Students commented that ChatGPT can help them improve their language skills by providing accurate reviews and improving the clarity, coherence, and correctness of their work, helping them convey their ideas effectively. This not only elevates the quality of their submissions, but also fosters and improves their writing skills. Furthermore, faculty explained that ChatGPT can benefit them significantly through language proofreading and editing services when preparing research papers, grant proposals, and scholarly publications. This driver may ensures that their academic work meets the highest standards, enhancing the likelihood of acceptance by prestigious journals and funding agencies.

Regarding problem-solving and data analysis, as some of the key drivers pushing students to use ChatGPT in education, all students explained that ChatGPT can be used as a problem-solving tool that conveys a transformative approach to education. This versatile AI-powered assistant provides immediate clarification of complicated or confusing concepts, and tackles challenging math problems in their coursework. Moreover, ChatGPT can perform various data analysis tasks, such as summarizing large volumes of text, identifying trends or recurring themes in conversations, categorizing information, solving math problems, and providing statistical or quantitative assessments of the data it processes. It empowers students to learn at their own pace and fosters independence by offering step-by-step solutions and explanations.

Another pivotal driver of using ChatGPT in education has been discussed by students and faculty in the context of test preparation. Students noted that the pressure of standardized tests and examinations can be overwhelming. ChatGPT steps in as an invaluable resource, offering targeted assistance in reviewing material, practicing problem-solving, and honing test-taking strategies. It provides students with the flexibility to access test preparation materials at any time, ensuring optimal readiness and boosting confidence. Moreover, faculty highlighted that ChatGPT is considered an instrumental tool in optimizing test preparation processes. They can use it to create practice questions, quizzes, and mock tests that align with the curriculum. Additionally, ChatGPT’s data-driven insights can help the faculty identify areas wherein students may need additional support or tailored test preparation resources, enabling educators to fine-tune their teaching strategies. 

Research support is considered as a crucial driver for using ChatGPT in education; it can serve as a guiding light, assisting them in formulating research questions, suggesting relevant sources, summarizing articles, and even generating preliminary research proposals. It empowers students and faculty with the tools needed to conduct more efficient and effective research, thereby elevating the quality of their academic work. Concept clarification related to the journey of understanding complex concepts can often be filled with confusion and uncertainty. ChatGPT can offer for students instant clarification and explanations on specific topics, and serves as a virtual tutor that can break down abstract ideas, provide real-world examples, and address specific queries, thus fostering enhanced comprehension and learning. Furthermore, ChatGPT may assist the faculty in explaining challenging concepts more effectively, ensuring that their teaching resonates with students, allowing them to stay up-to-date with the latest research and pedagogical techniques.

Concerning supplementary learning resources and adaptive learning, all students and faculty explained that ChatGPT can provide an extensive array of supplementary materials, including explanations, examples, practice questions, and recommended readings. These resources bolster traditional educational content, offering additional support for comprehension and skill development. Additionally, ChatGPT’s adaptive capabilities allow it to tailor responses and recommendations to each student’s unique needs and progress. By analyzing individual interactions, it can provide personalized guidance, adapt the difficulty level of questions, and offer targeted support in areas where a student may be struggling.

Another driver explained by the faculty is that ChatGPT can assist the faculty in assessing students’ level of competence, identifying areas of strength or weakness, and providing feedback or recommendations for improvement regarding their educational activities (e.g., quizzes, assignments, exams, discussions). Furthermore, ChatGPT can offer students scores or feedback, and may adapt subsequent interactions based on the assessment results.

#### 4.1.2. Consequences of Using ChatGPT for Academic Purposes in Higher Education

The incorporation of ChatGPT in education has brought both positive and negative consequences, shaping the learning approach of students. A summary of the key positive consequences is presented in Table 2. Concerning positive consequences, both students and faculty commented that ChatGPT is a remarkable timesaving tool. It provides immediate response, and they no longer need to spend excessive hours searching for answers or tackling complex concepts. Another two positive consequences explained by students concerned reducing anxiety and improving language skills. Students commented that assignments and homework make them feel anxious. ChatGPT steps in as a non-judgmental and readily available resource that allows students to practice and refine their language skills in a stress-free environment. It lowers the anxiety associated with language-related challenges and boosting students’ confidence in their ability to articulate their thoughts effectively. ChatGPT also serves as an indispensable tool for language learners. Its capacity to offer grammar suggestions, vocabulary enhancements, and coherent sentence structures aids students in honing their writing and communication skills.

Another positive aspect that has been explained by students and faculty is self-confidence. ChatGPT plays a key role in boosting self-confidence among students. When students receive prompt and accurate responses to their queries and challenges, they develop a sense of competence and mastery over the subject matter. Furthermore, ChatGPT’s support can help students tackle complex problems and assignments, making them feel more capable and confident in handling academic tasks. Regarding on-time submission, all faculty stated that ChatGPT can significantly improve time management skills, as students can allocate more time to researching, writing, and revising their work. With ChatGPT’s support, students are better equipped to meet deadlines without the stress of last-minute rushes, ultimately leading to a higher rate of on-time submissions. Moreover, the time saved by using ChatGPT for tasks like research and content generation allows students to focus on other aspects of their education, contributing to a more balanced and manageable workload. Another pivotal positive consequence pertains to the assistance and information provided by ChatGPT’s AI system to students for tasks and inquiries that are unrelated to their academic coursework or learning objectives, such as enrollment, course registration, scheduling, campus resources, financial aid, student services, and other logistical or procedural queries that students may have during their educational journey. ChatGPT can offer prompt and helpful responses to such administrative inquiries, simplifying students’ access to information and resources outside the realm of academia.

However, the rapid adoption of ChatGPT also presents negative consequences, which have been raised by education leaders/experts and faculty members (see Table 3). The two most prominent concerns related to using ChatGPT in education are the overreliance on AI in education, and academic integrity. While ChatGPT serves as an invaluable tool for learning, overreliance can potentially hinder the development of essential critical thinking and problem-solving skills. Students may become overly dependent on ChatGPT for answers, sacrificing their own exploration and analysis. With regard to academic integrity, ChatGPT can be misused by students for unethical purposes, such as plagiarism or cheating. When students rely on ChatGPT to generate assignments or answers without proper consideration of originality, it compromises the fundamental principles of academic honesty. This lack of integrity not only erodes the credibility of their educational achievements, but also diminishes the value of learning itself. Students may use ChatGPT to generate content that is not their own and present it as original work. Despite ChatGPT’s remarkable capabilities, it is not immune to challenges related to the lack of quality and accuracy in its responses. Like any AI system, ChatGPT relies on the data it was trained on, and these data can contain inaccuracies, biases, and inconsistencies. As a result, there are instances where ChatGPT may generate incorrect or misleading information, which can be problematic, especially in education or learning contexts. Additionally, the quality of ChatGPT’s responses can vary depending on the specificity and complexity of the query. 

Concerning learning outcomes, ChatGPT can have a negative influence on students’ social interaction in education when it becomes a primary source of learning and assistance. Relying on ChatGPT for educational support can reduce opportunities for face-to-face interactions between students and faculty. It may also discourage peer collaboration and limit the development of essential communication skills. In extreme cases, it could lead to a sense of social isolation, as students miss interpersonal communication resulting from traditional classroom interactions, which could negatively affect their learning experience. There were two more negative consequences cited by faculty and educational leaders: potential bias and student skill set. Potential bias represents a significant negative effect of using ChatGPT in education. All faculty and education leaders commented that ChatGPT’s responses are generated based on vast datasets, and if these datasets contain biases, they can be inadvertently perpetuated in ChatGPT’s interactions with students. This bias can manifest in various forms, including gender bias, racial bias, or cultural bias, and it can lead to unfair or discriminatory responses, reinforcing stereotypes or prejudices. This not only hinders the AI’s ability to provide equitable assistance, but also raises ethical concerns in educational settings.

Another pivotal negative consequence has been explained by students, faculty and educational leaders. The over-dependence on ChatGPT in education can have detrimental effects on students’ skill sets. While ChatGPT offers quick solutions and readily available information, it can inadvertently hinder the development of critical skills essential for lifelong learning. The excessive use of this AI tool can discourage independent thinking, problem-solving, and research skills, as students may prioritize convenience over intellectual engagement. Furthermore, ChatGPT’s assistance with writing and communication tasks may diminish students’ abilities to express themselves effectively, as they may become excessively dependent on AI-generated content.

#### 4.1.3. Recommendations for Mitigating the Negative Consequences of ChatGPT Use in Higher Education

There are several recommendations offered by faculty and education leaders in order to overcome the negative consequences of the misuse of ChatGPT for educational purposes. A summary of the key strategies or recommendations in terms of mitigating the negative consequences of ChatGPT use in education is presented in Table 4.

In terms of students’ overreliance on ChatGPT, faculty should establish and promote clear guidelines on the responsible use of ChatGPT. These guidelines can include defining the boundaries of AI assistance and learning in relation to the potential pitfalls of overreliance. They have to emphasize the importance of critical thinking and problem-solving skills as fundamental aspects of education. Encouraging students to question, analyze, and synthesize information independently fosters self-reliance and reduces dependency on AI tools like ChatGPT. Regarding academic integrity concerns such as intellectual property rights, cheating and plagiarism, students should receive training on responsible use and ethical guidelines, and only use AI for getting ideas and information, and in trying to illustrate them using their own writing skills. Moreover, educational institutions can invest in plagiarism detection tools that can identify AI-generated content and distinguish it from original work. Such tools can help maintain the integrity of academic assessments and uphold intellectual property rights. In order to combat students cheating using ChatGPT, proactive measures are essential. Students must understand the importance of academic honesty and avoid using ChatGPT to gain an unfair advantage. The faculty play a pivotal role in addressing this issue by designing assessments that are less accommodating of AI-generated content. They should focus on assignments that require critical thinking, problem-solving, and creative expression, tasks that go beyond the capabilities of AI and cannot be easily outsourced. Concerning the lack of quality and accuracy in ChatGPT, AI developers and organizations should continuously refine and improve the underlying AI models through rigorous testing and validation processes. This includes fine-tuning the training data to minimize biases and inaccuracies, and implementing mechanisms for regular updates to reflect evolving knowledge. Furthermore, the faculty should encourage students to cross-reference AI-generated information with trusted academic sources, foster a habit of discernment, and reinforce the importance of accuracy. Concerning learning outcomes, both educational institutions and faculty should encourage students to attend lectures and participate in class discussions to ensure they are comprehensively absorbing the subject matter. They should set specific learning goals and objectives for each study session, focusing on what they aim to achieve. Additionally, students should regularly reflect on their progress and seek feedback from faculty to identify areas that require improvement. 

In terms of the potential bias that results from using ChatGPT in education, students should take proactive steps, starting with diversifying their information sources and cross-verifying the information provided by ChatGPT from multiple reputable sources. They should be mindful of the possibility of bias and critically evaluate the content they receive, particularly in the context of sensitive or controversial topics. Regarding students’ skill set, students should approach ChatGPT as a valuable support tool rather than a crutch. It is essential to strike a balance between using AI assistance and honing essential skills. Independent research, critical thinking, and problem-solving exercises are important to graduates from higher education. Collaboration with peers and interactions with faculty remain crucial for skill development. They should seek feedback on their work, participate in class discussions, and ask questions to foster intellectual growth.

## 5. Discussion and Implications

ChatGPT has attracted the attention of key stakeholders in higher education institutions worldwide, due to its groundbreaking potential applicability in the realms of education, learning, and student support [18]. The current research confirms that stakeholders’ perceptions about using ChatGPT for academic purposes in higher education vary. Students tend to concentrate on the potential benefits of integrating ChatGPT into their educational activities, which supports the findings of previous research [3,9], whereas the faculty acknowledge both benefits and concerns related to using ChatGPT as an educational tool [3,8,9]. On the other hand, education experts/leaders were more cautious, and predominantly emphasized the key concerns and negative consequences of utilizing ChatGPT for academic purposes in education. 

The current research has outlined twelve key drivers of using ChatGPT in educational contexts. Consistent with several other studies, there are nine key drivers or benefits, which are quick response [4], ease of use [5], classroom and homework assistance [10], problem solving [3], data analysis [3,4], concept clarification [12], adaptive learning [3,5], assessment activities [15], and supplementary learning resource [4]. Furthermore, this research added other three drivers, mainly concerned with students and faculty, which are language proof-reading and editing, test preparation and research support. Ease of use and quick response are considered the most common drivers that have been cited by stakeholders, helping to enhance task optimization and efficiency within educational settings. Other common drivers pushing students to use ChatGPT in education are adaptive leaning, problem-solving and data analysis. ChatGPT allows adaptive learning through tailoring responses and suggestions according to the specific requirements and progress of each student. Through the analysis of individual interactions, it can offer personalized guidance, adjust the complexity of questions, and provide focused assistance in areas where a student may encounter difficulties. This aligns with the constructivist approach, as ChatGPT builds upon existing information by tailoring the necessary knowledge and resources [15]. This enhancement of learning is facilitated by capitalizing on prior knowledge to establish new associations and meanings that ultimately contribute to the acquisition of new knowledge. Concerning problem-solving and data analysis, ChatGPT can serve as a tool for data analysis and problem-solving, offering a transformative approach to education. Drawing upon the Constructivist Learning Theory, one crucial principle emphasizes that learners actively engage in the process of knowledge creation by exploring and uncovering the fundamental principles that form the basis of the concepts they are studying. Aligning with this approach, students would be encouraged to participate in observations, analyze data, and collaborate in solving problems within their learning process.

This research has confirmed that there are both positive and negative consequences attached to the use of ChatGPT in education, as explained by stakeholders. There are six positive and six negative consequences of using ChatGPT in education. The positive consequences include the greater saving of time [4], reduced anxiety [8], improved language skills [3], self-confidence [15], punctual submissions and non-academic support [3]. One of the most crucial positive consequences cited by students is non-academic support. The constructivist theory of learning underscores the significance of active learning, whereby learners engage actively in the learning process rather than passively receiving information. Another two positive consequences also highlighted by students related to reducing anxiety and improving their language skills. Students revealed that homework activities make them feel anxious. ChatGPT serves as a non-judgmental and easily accessible tool, affording students the opportunity to practice and enhance their language skills within a comfortable educational environment. It lowers the anxiety associated with language-related challenges, boosting students’ confidence in their ability to articulate their thoughts effectively.

However, the negative aspects cited by faculty and education leaders include overreliance, compromised academic integrity [11], lack of quality and accuracy [8], compromised learning outcomes [8], potential bias [3,4] and student skill set depletion [3]. The utilization of ChatGPT in education raises two primary concerns: an overreliance on AI in the educational process, and issues related to academic integrity. While ChatGPT has been proven to be an invaluable learning tool, an overreliance on it may potentially impede the development of critical thinking and problem-solving skills that are crucial for students. There is a risk that students might excessively rely on ChatGPT for answers, diminishing their own capacity for exploration and analysis. In terms of academic integrity, concerns arise due to the potential misuse of ChatGPT by students for unethical purposes, such as plagiarism of intellectual property or cheating. When students utilize ChatGPT to generate assignments or responses without proper attribution or originality, it undermines the fundamental principles of academic honesty and equitable evaluation. Another negative consequence cited by both faculty and education leaders concerns learning outcomes. The dependence on ChatGPT as a primary source of learning and assistance can potentially have adverse effects on students’ social interactions within the educational context. This dependence may lead to diminished opportunities for in-person interactions between students and faculty. Furthermore, it may discourage collaborative efforts among peers and curtail the cultivation of vital communication skills.

The above results have some practical and theoretical implications. With regard to practical implications, the current research has shown that the use of ChatGPT for learning purposes should be the focus of more attention from key stakeholders. It could be used with caution, to avoid the realization of its negative consequences. The current research provides a set of recommendations to mitigate these negative consequences related to ChatGPT usage in higher education. Higher education institutions should establish and promote clear guidelines on the responsible use of ChatGPT. They should provide students with training on responsible use and ethical guidelines. Students should understand that they could use AI for developing ideas, deriving information, and developing their own writing skills. Moreover, educational institutions should invest in plagiarism detection tools that can identify AI-generated content and distinguish it from original work. Faculty should encourage students to cross-reference AI-generated information with trusted academic sources, so as to foster a habit of discernment and reinforce the importance of accuracy. Students should take proactive steps, starting with diversifying their information sources and cross-verifying the information provided by ChatGPT with multiple reputable sources. Furthermore, they should strike a balance between using AI assistance and developing their skills. Additionally, collaborations with their peers and interactions with faculty remain crucial for skill development. Also, students should seek feedback on their work, participate in class discussions, and ask questions to foster intellectual growth. Scholars, on other hand, should neither support nor reject the use of ChatGPT for learning or other academic purposes without understanding both the positive and the negative consequences. The long-lasting consequences in relation to students’ learning outcomes, as well as ethical considerations and the sustainability of higher education institutions, have to be considered when examining the integration of any AI tool used for learning in higher education. The perspectives of various stakeholders must be considered in order to develop a better understating of this issue. 

## 6. Conclusions

ChatGPT is a transformative and multifaceted AI tool that serves as an adaptable resource for both students and faculty in dynamic learning environments. The current research has shown that there are twelve key drivers motivating students towards using it in their learning. These key drivers are quick response, ease of use, classroom and homework assistance, problem solving, data analysis, concept clarification, adaptive learning, assessment activity and supplementary learning resource provision, language proof-reading and editing, test preparation and research support. The research has also shown that there are both positive and negative consequences attached to the use of ChatGPT in education, as explained by stakeholders—six positive and six negative. The positive consequences include time saving, reduced anxiety, improving language skills, self-confidence, punctual submission and non-academic support. However, the negative areas of concern, which are mainly cited by faculty and education experts/leaders, include overreliance, the loss of academic integrity, lack of quality and accuracy, damaged learning outcomes, potential bias and deteriorated student skill sets. The research thus provides some recommendations for overcoming the negative consequences. The key strategy involves higher education institutions establishing a clear policy and guidelines regarding the use of ChatGPT for academic purposes in higher education. ChatGPT could be used as a supporting tool to enhance students’ learning and their understanding of specific topics and concepts.

This research used a qualitative approach with a sample of students, faculty and education experts/leaders in public KSA universities. Therefore, the results could not be generalized to other contexts without further testing. Future research avenues could include quantitatively testing the key drivers or factors that shape students’ usage of ChatGPT for learning and other related academic purposes in higher education. In addition, using ChatGPT has long-term consequences, both positive and negative. However, students misusing this tool could have an impact on the educational process, which should also be addressed in future research. One future research avenue could be a comparison between male and female students in terms of their use of AI, and its relationship with their academic performance. Moreover, future studies could examine the factors from the faculty’s perspective. Other theoretical framework, such as TAM or TPB, could be adopted for interpreting the key drivers of academic-related use in higher education.

## Figures and Tables

**Table 1:** Key drivers of ChatGPT use for academic purposes in higher education.

Drivers	Description
Quick Response (ST, FC, EDLE) *	ChatGPT can provide immediate feedback on assignments, questions, and exercises.
Ease of Use (ST, FC, EDLE) *	ChatGPT offers a smooth, simple, friendly and understandable interface for users to input queries, receive responses, and navigate its functionalities.
Classroom and Homework Assistance (ST, FC) *	ChatGPT can facilitate teaching and studying by explaining specific topics.
Language Proof Reading and Editing (ST, FC) *	It could correct lanague, such as via spelling, grammar, punctuation, and syntax errors, in written assignments or projects.
Problem Solving (ST) *	The capability of ChatGPT to assist students in resolving issues, answering questions, or finding solutions to various challenges or inquiries.
Data Analysis (ST) *	The capability of ChatGPT to assist students in complex textual data inteprretaion, tackling analytical challenges, and solving math problems and puzzles.
Test Preparation (ST, FC) *	ChatGPT can generate practice questions, review key concepts, and test knowledge on specific subjects.
Research Support (ST, FC) *	ChatGPT can assist in conducting research by providing relevant information, suggesting sources, and helping refine research questions.
Concept Clarification (ST, FC) *	ChatGPT can offer clear explanations and examples to aid in understanding unknown concepts.
Supplementary Learning Resource (ST, FC) *	ChatGPT can provide additional educational material, information, or assistance to enhance a student’s understanding of a subject or topic.
Adaptive Learning (ST, FC) *	ChatGPT can tailor its responses and recommendations based on student needs, progress, and preferences.
Assessment Activities (FC) *	ChatGPT can assist faculty in activities, assess students‘ levels of competence, identify areas of strength or weakness, and provide feedback or recommendations for improvement.

* Codes of participants who commented on this point (ST = students; FC = faculty; EDLE = education leaders/experts).

**Table 2:** Positive consequences of ChatGPT use for academic purposes in education.

Consequences	Description
Time Saving (ST, FC) *	ChatGPT can provide quick answers and explanations, saving students and faculty time when researching or studying. This can be especially valuable when students have tight deadlines or the faculty have limited time to do their work.
Reduced Anxiety (ST) *	For students who are hesitant to ask questions in a classroom setting, ChatGPT provides a low-pressure environment where they can seek clarification and assistance without fear of judgment.
Improving Language Skills (ST) *	ChatGPT could help students improve their language skills through providing immediate grammar, vocabulary, and pronunciation checks, making it a valuable tool for education.
Self Confidence (ST, FC) *	ChatGPT empowers students by providing them with valuable tools and resources that enhance their learning experience, ultimately boosting their self-confidence.
On-Time Submission (FC) *	ChatGPT can facilitate efficient time management, reduce procrastination, and enhance students’ puctuality in the submission of their educational activities.
Non-Academic Support (ST) *	ChatGPT can assist and guide students in various administrative matters to enhance their learning experience.

* Codes of participants who commented on this point (ST = students; FC = faculty; EDLE = education leaders/experts).

**Table 3:** Negative consequences of ChatGPT use for academic purposes in education.

Consequences	Description
Overreliance (FC, EDLE) *	Excessive dependence on ChatGPT can lead to students relying on it for answers without truly understanding the material. This can hinder critical thinking and problem-solving skills.
Academic Integrity (FC, EDLE) *	The misuse of information and knowledge generated by ChatGPT may result negatively in copyright violations.
Lack of Quality and Accuracy (FC, EDLE) *	While ChatGPT can provide quick responses, the accuracy and quality of the information can vary. Incorrect or misleading answers or information may negatively impact learning outcomes.
Learning Outcomes (FC, EDLE) *	The use of ChatGPT has a negative impact on educational support and may reduce social interaction between students and faculty, as well as affecting their learning experience.
Potnetial Bais (FC, EDLE) *	ChatGPT may generate responses or recommendations that reflect unintentional prejudices or imbalances.
Student Skill Set (FC, EDLE) *	Excessive reliance on ChatGPT may cause students to habitually turn to ChatGPT for answers, as well as hindering the development of students’ essential skills.

* Codes of participants who commented on this point (ST = students; FC = faculty; EDLE = education leaders/experts).

**Table 4:** Recommendations for overcoming negative consequences of ChatGPT use for academic purposes in education.

Consequences	Recommendations
Overreliance (FC, EDLE)	▪ Educational institutions should establish clear guidelines on the resposible use of ChatGPT in academic settings as a supproting tool. ▪ Faculty should encourage their students to reponsibly use ChatGPT, while they ensure the development of their critical thinking and problem-solving skills.
Academic Integrity (FC, EDLE)	▪ Responsible use and ethical guidelines are crucial to mitigate potential negative effects on intellectual property. ▪ Students should be trained in academic honesty and the use of resource, e.g., ChatGPT, in learning. ▪ Students should use ChatGPT and other electronic resources to support heir learning, not as a primary leaning tool. ▪ The faculty should design assessments that are less accomodating of AI-generated content.
Lack of Quality and Accuracy (FC, EDLE)	▪ Caution should be maintined while dealing with information generated by ChatGPT. ▪ AI developers and organizations should continuously refine and improve the underlying AI models through rigorous testing and validation processes. ▪ The faculty should encourage students to mantain caution and verify the accuracy of the information generated by ChatGPT before relying on it for their studies.
Learning Outcomes (FC, EDLE)	▪ Institutions should use techology to supplemt face-to-face education and not as a replacement for face-to-face education.
Potnetial Bias (FC, EDLE)	▪ Students should seek information from a variety of sources, including textbooks, academic journals, and human experts. This ensures a more comprehensive and balanced learning approach. ▪ Faculty should engage in critical thinking activities during the teaching and learning process.
Student Skill Set (FC, EDLE)	▪ Students should use ChatGPT as a supplementary resource rather than a primary source of information and solutions. ▪ Students should engage in activities that promote various skills such as critical thinking, problem-solving, and analysis.

Codes of participants who commented on this point (ST = students; FC = faculty; EDLE = education leaders/experts).

## Data Availability

The data from interviews could be shared with researchers who meet eligibility criteria after consent is given by the KFU Ethical Committee.
